# Body and mind: how obesity triggers neuropsychiatric and neurodegenerative disorders

**DOI:** 10.3389/fpsyt.2024.1524555

**Published:** 2025-01-07

**Authors:** Claudio Pirozzi, Nicola Opallo, Filomena Del Piano, Stefania Melini, Adriano Lama

**Affiliations:** ^1^ Department of Pharmacy, School of Medicine, University of Naples Federico II, Naples, Italy; ^2^ Department of Veterinary Medicine and Animal Productions, University of Naples Federico II, Naples, Italy

**Keywords:** Alzheimer's disease, Parkinson's disease, mood disorders, gut-brain axis, metabolic dysfunction, gut microbiota

## Introduction

Obesity has emerged as a significant health concern, particularly affecting young people worldwide. Its prevalence extends beyond Westernized countries and has been projected to rise from 107.7 million obese children and adolescents in 2015 to 254 million by 2030 (1). This metabolic disorder poses severe consequences for healthcare systems globally, as childhood obesity often persists into adulthood (2). Unlike other diseases, obesity is a pathological condition that renders individuals more susceptible to various disorders, including metabolic syndrome, cardiovascular disease, non-alcoholic fatty liver disease, and cancer (3).

Metabolic dysfunctions disrupt the structural and functional systems of humans, including the central nervous system (CNS) (4). The hypothalamus, a pivotal region situated between the CNS and periphery, serves as the central control center for energy homeostasis, body temperature, food intake, and other essential functions (5). Consuming a diet high in calories, particularly carbohydrates and lipids, triggers a vicious cycle of hyperactivation of central immune cells and neuroinflammatory mediators within the hypothalamus, resulting in widespread effects (6). Consequently, alterations in this key brain region led to impairments in all related neuronal circuits of other brain areas, including the mesolimbic dopamine (DA) system, hippocampus, nucleus accumbens, striatum, and cortex, which are primarily associated with functions such as cognition and mood regulation.

The gut microbiota plays a pivotal role in the pathophysiology of neuropsychiatric and neurodegenerative disorders, particularly in the context of obesity. The gut-brain axis mediates this relationship (7). Obesity-induced peripheral alterations influence brain function by enhancing neuroinflammation, altering neurotransmitter synthesis, and impairing insulin signaling (7). These mechanisms have been associated with elevated risks of depression, anxiety, cognitive decline, and neurodegenerative diseases in obese individuals (8).

Specifically, Proteobacteria and Cyanobacteria are overrepresented in the gut microbiota of obese patients (9, 10). The dysregulation of metabolites associated with these bacteria may contribute to systemic inflammation and oxidative damage, indirectly affecting neuroinflammatory pathways relevant to neuropsychiatric and neurodegenerative diseases (11, 12).

## Obesity and neuropsychiatric disorders

Obesity and mood disorders are intricately interconnected, and their pathological mechanisms share numerous characteristics (13). At the CNS level, obesity-related detrimental factors compromised the integrity of the blood-brain barrier (BBB), a pivotal barrier that prevents the entry of detrimental substances into the brain (14). The BBB is a sophisticated and highly specialized biological construct, characterized by its selective permeability and protective role within the CNS. Obesity leads to structural and functional alterations of the BBB, including increased permeability, altered transport mechanisms, and inflammatory responses (14). Consistently, our findings indicate that the disruption of the blood-brain barrier (BBB), induced by a high-fat diet (HFD) and evidenced by albumin extravasation in the hippocampus of obese mice, represents a critical mechanism that contribute to the pathogenesis of mood disorders (15). In this condition, peripheral signals via the gut-microbiota-brain axis can impact CNS activity, both negatively and positively (7). Obesity-induced metainflammation, characterized by increased levels of pro-inflammatory mediators, activates astrogliosis and microgliosis, leading to neuroinflammation (16). The role of microglia, the resident immune cells in the CNS, is particularly relevant in the context of neuroinflammation and mood disorders (17). Under normal conditions, microglia maintain homeostasis by regulating synaptic pruning and clearing cellular debris. However, in response to chronic stress or obesity-related inflammation, microglia can become activated and release inflammatory mediators that lead to neurotoxicity (18). The hyperactivation of central immune cells blocks the pivotal machinery responsible for neurogenesis, the process of neuronal renewal (19). Indeed, activated microglia reduce neurogenesis by suppressing neuronal stem cell proliferation, increasing apoptosis of neuronal progenitor cells, and decreasing survival of newly developing neurons and their integration into existing neuronal circuits (20). Furthermore, long-term consumption of a Western-style HFD, characterized by low fiber content, may lead to a substantial reduction in short-chain fatty acids, endogenous molecules with notable anti-inflammatory and neurogenesis-promoting properties (21). Our studies demonstrated that HFD feeding induced depressive- and anxiety-like behavior associated with intestinal dysbiosis, with the proliferation of inflammatory-related microbes, and alteration of gut tryptophan metabolite pathway (22). The production of toxic metabolites of tryptophan, such as quinolinic and kynurenic acid, may severally impact CNS homeostasis (23). The neurobiological alterations induced by a HFD encompass impairments in reward circuitry, diminished serotonin (5-HT) and DA levels, and augmented hypothalamic-pituitary-adrenal axis (HPA) activity, a critical component of the body’s stress response system (24). In particular, neuroinflammation is known to disrupt the balance of neurotransmitters essential for mood regulation, particularly 5-HT and gamma-aminobutyric acid (GABA). Elevated levels of interleukin (IL)-6 and tumor necrosis factor (TNF)-α have been associated with dysregulation of serotonergic and GABAergic systems, which may exacerbate mood disorders symptoms in individuals with obesity (25). These conditions can serve as the etiological basis for depressive and anxiety phenotypes.

Given the growing and intricate relationship between obesity and neuropsychiatric disorders, recent evidence also highlights shared mechanistic targets between the two conditions. For instance, peroxisome proliferator-activated receptor (PPAR)-α, whose role in lipid metabolism of various tissues is well-established in clinical therapy, has recently been recognized as an endogenous tranquilizer of mood disorders caused by dysmetabolism (26). PPAR-α is widely distributed across the amygdala, prefrontal cortex, thalamic nuclei, nucleus accumbens, ventral tegmental area (VTA), and basal ganglia (27). Moreover, research by Jiang et al. (28) demonstrated that the PPAR-α agonist WY14643 ameliorated depressive-like behaviors in mice, an effect attributed to the activation of the Brain-Derived Neurotrophic Factor signaling pathway.

## The mutual interrelatedness between obesity and neurodegenerative diseases

Neurodegenerative diseases and obesity are increasingly recognized to have a complex, bidirectional relationship (29). Obesity, particularly in midlife, has been associated with an elevated risk of developing several neurodegenerative disorders, such as Alzheimer’s disease (AD), Parkinson’s disease (PD), and other forms of cognitive decline (30). This correlation is mediated by a combination of metabolic, inflammatory, and hormonal factors that affect brain health (31). These factors share biological mechanisms, including chronic inflammation, insulin resistance, hormonal changes (leptin and adipokines), oxidative stress and related mitochondrial dysfunction, and altered gut microbiota (32). Metainflammation, a key feature in many neurodegenerative diseases induced by dysmetabolism, impairs the BBB and triggers neuroinflammation (15). Neuroinflammation, in turn, accelerates neuronal damage and contributes to disease progression. Consistently, obesity increases the production of free radicals and decreases the antioxidant defenses, leading to oxidative stress (33). This process, coupled with impaired mitochondrial function, damages cellular structures, including lipids, proteins, and DNA, accelerating the degeneration of neurons and other brain cells (34).

## Obesity and Alzheimer’s disease

Emerging research underscores the role of metabolic disorders, particularly obesity, in the etiology of AD, even if aging is the primary risk factor for AD (35). Therefore, obesity significantly correlates with cognitive dysfunction and is a substantial risk factor for developing AD (36). In this context, the excessive body weight can lead to inflammatory processes and metabolic derangements that may increase the risk of amyloid β (Aβ) accumulation in the brain (37). The connection between obesity and AD extends beyond Aβ deposition; both conditions are associated with a chronic low-grade inflammatory state, or metainflammation, characterized by oxidative stress and increased production of reactive oxygen species (38). These factors contribute to neuronal damage and cognitive decline (39). Furthermore, HFD-linked obesity has been observed to induce mitochondrial dysfunction and impair antioxidant defenses, impacting brain health (40).

Notably, the DA system, particularly the VTA, is involved in both obesity and cognitive function. Different types of HFDs alter this system, potentially affecting reward perceptions related to food intake (41).

Insulin signaling influences DA transporter activity, establishing a connection between metabolic health and cognitive outcomes (42). This connection appears to involve increased expression of the dopamine-degrading enzymes MAO-A and MAO-B, which reduces dopamine (DA) signaling (43). Furthermore, DA has been demonstrated to reduce microglial inflammation, potentially impacting the progression of neurodegenerative processes in AD (44). This intricate interplay implies that obesity not only increases the risk of developing AD but also contributes to its pathophysiology through mechanisms involving inflammation and metabolic dysfunction primarily affecting adipose tissue.

## Parkinson’s disease and obesity: exploring the connection

While PD is traditionally associated with genetic factors and environmental toxins, emerging research indicates that obesity may also contribute to both the development and progression of PD (29). Specifically, several studies have suggested that obesity, particularly in midlife, may increase the risk of developing PD later in life. For instance, individuals with a body mass index in the obese range at midlife exhibited a higher likelihood of developing PD in their later years (45).

Obesity has been linked to the worsening of both motor and non-motor symptoms of PD (46). It can impact the motor system by causing stiffness, decreasing flexibility, and hindering physical activity, which is already a significant challenge for individuals with PD. Additionally, obesity, particularly in older adults, has been linked to an increased risk of cognitive decline (29). The relationship between obesity and cognitive dysfunction in PD may be attributed to factors such as chronic inflammation, vascular health, and metabolic dysfunction, which can all contribute to worsen brain health in individuals with PD (47). Obesity leads to an increased systemic inflammation, during which adipocytes release pro-inflammatory cytokines such as TNF-α, IL-6, and C-reactive protein (48). Lipid overnutrition and subsequent metainflammation can have a direct impact on the brain, exacerbating neuroinflammation, which is a key player in the progression of PD. As it contributes to the degeneration of dopaminergic neurons, neuroinflammation plays a crucial role in the development of PD. In the brain, microglia are activated during neuroinflammation as a key mechanism in PD (49). Since microglia release neurotoxic substances that promote neuronal damage, obesity-induced inflammation may further exacerbate this microglial activation, accelerating the progression of PD (50).

At the molecular level, obesity has been demonstrated to influence brain circuits that regulate DA, potentially contributing to both the onset and progression of PD (51). Notably, leptin, primarily produced by adipocytes, plays a role in the regulation of the brain’s DA system (52). Research suggests that leptin resistance, as a hallmark of obesity, can impair DA release and signal transduction, potentially exacerbating the symptoms of PD (53).

## Conclusions

**Figure 1:**
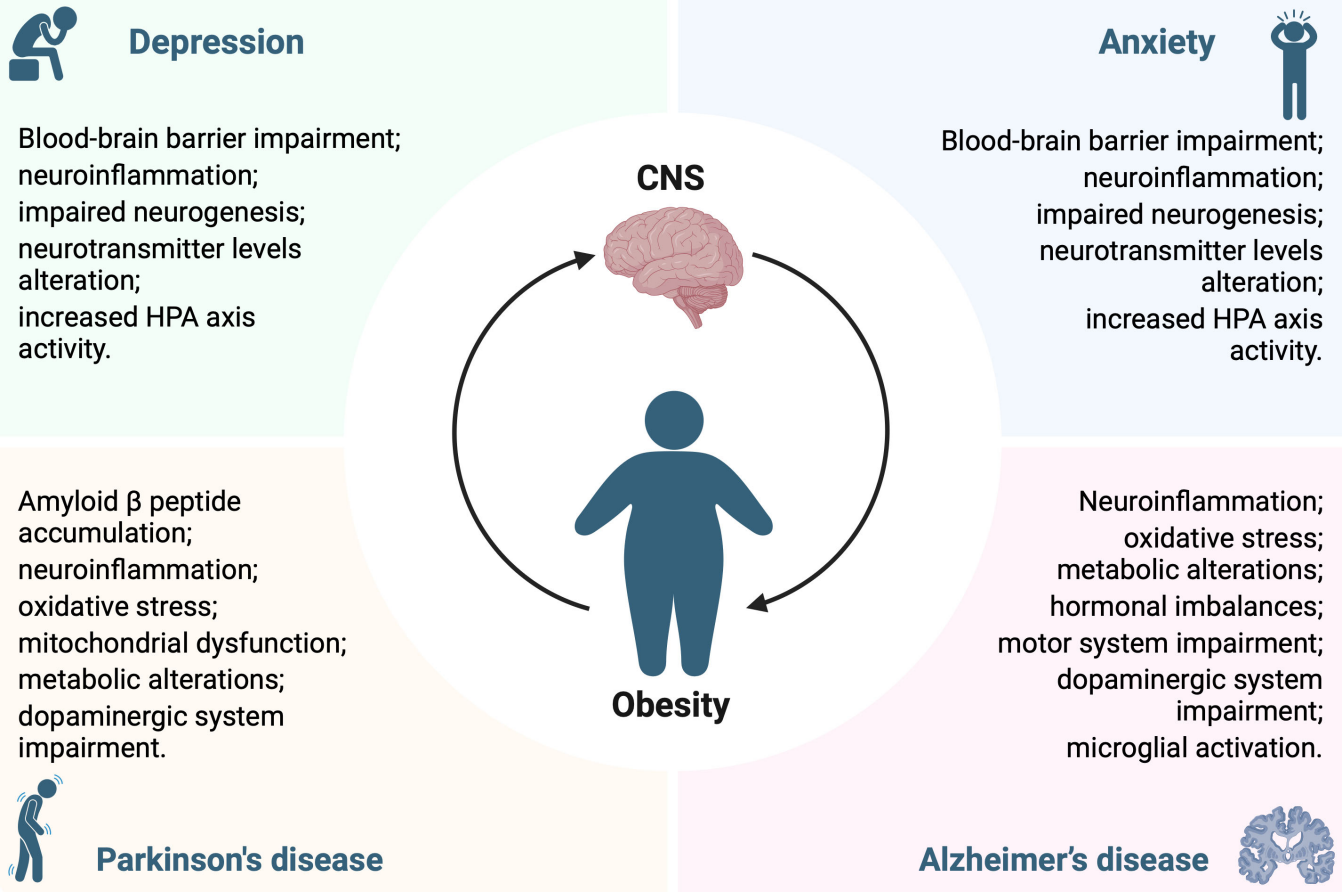
Pathological mechanisms underlying the interconnection between obesity and central nervous system disorders.

The purpose of this opinion is to draw attention to the obesity-driven neuropsychiatric and neurodegenerative disorders, investigating how to address the “pandemic of wellness.” Identifying causal factors and their interrelationships in the development of obesity and its comorbidities, although a complex and intricate task, is crucial to prevent or counteract neuropsychiatric and neurodegenerative disorders caused by gluco-lipid dysmetabolism. The scientific community has made significant progress in understanding the pathogenic mechanisms involved in obesity-induced CNS disorders (**Figure 1**), but there is still much to accomplish. Furthermore, contemplating and possibly targeting the metabolic derangements due to obesity could be critical in developing innovative therapeutic strategies to counteract obesity-related central disorders, emphasizing the necessity for a comprehensive approach to brain health which takes into account the impact of metabolic disorders.
